# Development of Atypical Neuroleptic Malignant Syndrome After Treatment of Cocaine Intoxication: A Case Report and Literature Review

**DOI:** 10.7759/cureus.35538

**Published:** 2023-02-27

**Authors:** Maxwell Miller, Alisa Zezetko, Ritvij Satodiya

**Affiliations:** 1 Psychiatry, East Carolina University, Greenville, USA; 2 Child and Adolescent Psychiatry, New York University Grossman School of Medicine, New York, USA

**Keywords:** dysregulated dopamine signaling, dysautonomia, cocaine intoxication, atypical neuroleptic malignant syndrome, neuroleptic malignant syndrome (nms)

## Abstract

Neuroleptic malignant syndrome (NMS) is a life-threatening condition classically associated with the use of antipsychotic medications. NMS commonly presents with initial mental status changes, followed by muscle rigidity, fever, and eventual dysautonomia. Cocaine intoxication can present with symptoms that are very similar to those found in NMS, making differentiating the two disorders challenging. We present the case of a 28-year-old female with a history of cocaine use disorder who presented with acute cocaine intoxication. She had severe agitation associated with her intoxication, requiring the use of antipsychotic medications. Subsequently, she developed atypical NMS from abrupt dopamine withdrawal after receiving the antipsychotics. Although overlapping dopamine pathways between cocaine use and NMS could deter one from this practice and guidelines recommend against it, antipsychotics are routinely used in the emergency setting for cocaine-associated agitation. This case highlights the need for a more standardized treatment protocol, provides an explanation of why treating cocaine intoxication with antipsychotics is inappropriate, and suggests that chronic cocaine users may be more prone to NMS in this scenario. Furthermore, this is a unique case because it describes atypical NMS in the context of cocaine intoxication, chronic cocaine use, and administration of antipsychotics to an antipsychotic-naïve patient.

## Introduction

Neuroleptic malignant syndrome (NMS) is a life-threatening emergency that is classically associated with the use of antipsychotic medications, which is thought to occur from a sudden loss of dopamine D2 receptor (D2R) activity, either by dopamine withdrawal or receptor blockade [1]. Antipsychotic medications are a class of psychotropic medications that are frequently used in the management of psychiatric conditions as well as psychiatric emergencies such as psychosis and agitation. Antipsychotics exert their primary therapeutic effect through dopamine D2R blockade.

The diagnostic criteria for NMS per the Diagnostic and Statistical Manual of Mental Disorders, Fifth Edition (DSM-5), requires three major criteria be met, including severe muscle rigidity, fever, exposure to a dopamine-blocking agent, and two minor criteria including diaphoresis, dysphagia, tremor, altered level of consciousness, incontinence, mutism, tachycardia, elevated or labile blood pressure, leukocytosis, and elevated creatinine phosphokinase (CPK) [1]. NMS is considered atypical when only three of the four cardinal features are present, which include fever, altered mental status (AMS), autonomic dysfunction, or muscle rigidity [2]. Initial symptoms in NMS are mental status changes, followed by muscular rigidity, fever, and finally dysautonomia in 70% of cases [3]. The onset of NMS symptoms from antipsychotic exposure is variable, with 16% of cases occurring within 24 hours, 66% of cases within seven days, and nearly all cases within 30 days [4]. Because several other syndromes can result in similar symptoms, brain imaging, lumbar puncture (LP), and electroencephalogram (EEG) should be obtained to exclude organic causes [2]. Treatment for NMS includes bromocriptine, dantrolene, benzodiazepines, and termination of all antipsychotics [1,5].

Cocaine, a psychostimulant, is believed to mediate its primary effects of euphoria and addiction by increasing dopamine activity at both the dopamine D1 receptor (D1R) and D2R by dopamine transporter inhibition [6]. It also affects the sympathetic nervous system by increasing the accumulation of monoamines such as dopamine, serotonin, and norepinephrine and the cardiovascular system by increasing alpha- and beta-1-adrenoreceptor activity, resulting in increased cardiac contractility [6]. Intoxication syndromes from drugs of abuse such as cocaine can therefore easily be confused with NMS due to significant symptom overlap including hyperthermia, mental status changes, and autonomic dysfunction [5]. The common clinical findings for NMS and cocaine intoxication are summarized in the Appendices.

Both cocaine and antipsychotics primarily involve the same dopaminergic pathways as those involved in NMS: the D2R hypothalamic, mesolimbic, striatum, and nigrostriatal pathways [7-9]. Antipsychotics, in theory, could block cocaine’s pro-dopamine effect, which may explain why it is thought to be more effective in combination with lorazepam for cocaine-associated agitation [6]. However, in a 2012 consensus statement by the American Association for Emergency Psychiatry, the use of antipsychotics for agitation secondary to stimulant use should be reserved for the few patients who present with psychotic symptoms, which typically only occur in chronic amphetamine users [10].

We postulate that the risk of NMS was higher in our patient with chronic cocaine use, who presented with acute intoxication with mental status changes requiring the use of antipsychotics. Furthermore, this case magnifies the problematic clinical scenario of differentiating NMS from cocaine intoxication, particularly in the setting of ongoing antipsychotic administration despite clinical deterioration, which is what occurred to our patient.

## Case presentation

A 28-year-old female with chronic cocaine use disorder and bipolar I disorder presented to an outside emergency department (ED) via emergency medical services (EMS) for severe agitation and erratic behavior at home in the context of a cocaine overdose. The patient was not taking any psychotropic medications, and she was chronically nonadherent to her prescribed medication regimen, which included lithium and clonazepam, and she had no prior history of treatment with any antipsychotic medications. Historically, she had not previously been treated with antipsychotic medications. In addition, she had a history of chronic viral hepatitis C as well as remote COVID-19 infection. Her family history was noncontributory, and her psychosocial history was significant for unemployment and her current residence with her mother. Her initial laboratory workup was negative for leukocytosis, anemia, and renal dysfunction, and her urine drug screen was positive for cocaine. She was admitted for acute cocaine intoxication in the setting of known chronic cocaine use.

On her first day in the ED, she was severely agitated, combative with staff, and unresponsive to verbal de-escalation. She received one dose of haloperidol 5 mg intravenous (IV), but it was not effective in controlling her symptoms. She was subsequently admitted to the medical floor for further observation and treatment. She was started on quetiapine 100 mg every eight hours orally and received two doses.

On the second day of her admission, quetiapine was increased to 150 mg every eight hours, and on day 3, given her escalating agitation and aggression, she was treated with several other antipsychotics on an as-needed basis. As a result, during her first 72 hours in the hospital, she received olanzapine 15 mg intramuscular (IM), haloperidol 15 mg intravenous (IV), ziprasidone 20 mg IM, and quetiapine 650 mg orally.

On her fourth day in the hospital, she was noted to have altered mental status, an elevated temperature of 107.2°F, and leukocytosis with a white blood cell count of 22.63 thousand per cubic milliliter (k/uL). Differential diagnoses included infectious causes such as septic shock, meningitis, and encephalitis as well as neurological causes such as seizures, neuro-storming in the context of cocaine intoxication, and NMS due to recent exposure to antipsychotic medications. Computed tomography scan and magnetic resonance imaging of the head and cervical spine both returned negative for intracranial hemorrhage, stroke, brain mass, or other relevant intracranial or cervical spine findings. She was started on piperacillin-tazobactam and vancomycin for empiric antibiotic coverage, but both were later discontinued after blood and urine cultures returned negative. Despite the absence of muscular rigidity, a CPK level was ordered on day 4 of her hospitalization due to concern for NMS, and it was found to be elevated at 3,799 international units per liter (IU/L). At this time, all antipsychotics were discontinued. She was treated with bromocriptine 2.5 mg orally twice daily and lorazepam 2 mg IV every six hours for possible atypical NMS. On the new regimen of bromocriptine and lorazepam, her fever improved, but her mentation continued to deteriorate.

On her fifth day of hospitalization, the neurology team was consulted for further recommendations in the setting of suspected atypical NMS given her recent antipsychotic exposure. Neurology agreed that her symptom onset and progression favored a diagnosis of atypical NMS and recommended LP and EEG to complete the workup for any organic etiology. They also advised giving one dose of dantrolene 1 mg/kg IV and continuing with scheduled bromocriptine and lorazepam. Later that day, she was noted to have a low oxygen saturation (SpO2) of 80% on room air, and supplemental oxygen was started. The primary team suspected that this sudden change was secondary to respiratory aspiration. Because of only intermittent compliance with keeping her oxygen equipment in place despite frequent redirection, her SpO2 continued to drop. In this setting, her oxygen requirements progressed to seven liters, and the results of arterial blood gas (ABG) indicated hypoxemic respiratory alkalosis with metabolic compensation. Due to impending respiratory failure, the decision was made to intubate the patient on the evening of hospital day number 5, and she was subsequently transferred to a regional level I trauma center intensive care unit (ICU) for a higher level of care.

**Figure 1:**
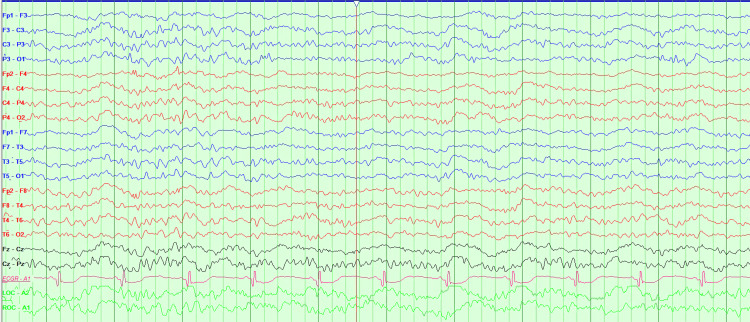
EEG showing diffuse slowing without any epileptiform activity captured EEG: electroencephalogram

Upon ICU admission, bromocriptine and lorazepam were both continued, and further neurologic workup was completed with LP and EEG. Her LP results were negative for any infectious or autoimmune etiologies; CSF culture, cytology, encephalitis, meningitis panels, herpes simplex virus, and anti-N-methyl-D-aspartate (NMDA) receptor antibodies were negative. Serum studies for antinuclear antibody (ANA) with reflex testing for antineutrophil cytoplasmic antibodies (ANCAs), as well as perinuclear antineutrophil cytoplasmic antibodies (p-ANCAs), were negative for autoimmune vasculitis. A routine EEG revealed diffuse slowing without any epileptiform activity captured, and these findings were suspected to be representative of her known encephalopathy requiring no additional neurological workup (Figure 1).

On her second day in the ICU, her fever subsided, and her CPK levels downtrend to less than 2,000 IU/L. She began following commands by the end of that day resulting in the discontinuation of both bromocriptine and lorazepam. On her third day in the ICU, she was successfully extubated and was determined to be medically stable for transfer to the general medical ward. The progression of her NMS from onset to resolution is summarized in the Appendices.

While in the general medical ward, she exhibited selective mutism. This was initially concerning for catatonia, and psychiatry was asked to evaluate the patient. Her score on Bush-Francis Catatonia Rating Scale was 2. Nevertheless, psychiatry recommended one dose of lorazepam 2 mg IV with an assessment before and after administration, and no improvement was noted after the administration of lorazepam. She was subsequently observed speaking to certain staff members and not to others throughout the remainder of her hospitalization, and her symptoms were determined to be volitional in nature as opposed to originating from any acute psychiatric illness. She was discharged home with recommendations for close outpatient psychiatric follow-up, substance abuse counseling, and instructions to avoid any antipsychotic medications until her outpatient appointment.

## Discussion

Studies involving brain imaging of cocaine-addicted human subjects with repeated exposure to cocaine revealed dysregulated dopamine release as well as reduced receptor availability in the striatum and several other brain regions [11]. These findings, coupled with the findings from neuroimaging studies involving subjects with repeated cocaine exposure, NMS, and exposure to antipsychotics, support the theoretical risk of NMS when treating cocaine intoxication with antipsychotics, particularly if they are chronic users [7-9,11,12]. In addition to the explanation for this theoretical risk, there are numerous cases in the literature that describe this process in clinical terms. However, none describe atypical NMS caused by cocaine withdrawal, in the setting of chronic cocaine use, in an antipsychotic-naïve patient after an extensive review of the literature.

One case series involving recreational cocaine users suggested that rapid death from sudden cocaine withdrawal shared many features found in traditional NMS with the notable exception of muscular rigidity in the majority of cases [13]. A comparative study of 166 psychiatric patients with and without substance use history treated with antipsychotics revealed that four or 5.1% of cocaine users developed NMS compared to none of the non-cocaine users [14]. One report described a case of NMS with lead-pipe rigidity in the setting of recent cocaine use by a chronic cocaine user with long-term antipsychotic treatment [15]. Another case of classic NMS was due to cocaine use that occurred in the absence of antipsychotic medications [16]. A third case reported NMS with muscular rigidity in the setting of antipsychotic use in a chronic cocaine user, who did not have any recent cocaine use [17].

NMS has a wide spectrum of presentations and can be extremely difficult to diagnose by even the most expert clinicians [3,18]. NMS classically presents with lead-pipe rigidity and mental status changes. However, this pair of findings was only present in 82.4% of 340 clinical reports assessing the four most common symptoms, and fever and autonomic dysfunction were present in 70.5% of the cases [18]. Similarly, there are cases of atypical presentations of NMS without elevated CPK or muscular rigidity [13,19]. Thus, NMS is a diagnostic challenge, which is unfortunately associated with a mortality rate of 20%-30% with death most often due to respiratory failure in the absence of early treatment [20].

We believe that our patient had NMS, which started and had been developing since her initial day of presentation, closely following the clinical course seen in 70% of NMS cases [3], and that abrupt dopamine withdrawal after treatment with antipsychotics on presentation caused her to develop NMS. Her clinical deterioration from the outset of her hospitalization was the earliest indicator of her NMS, and had the differential diagnosis been expanded in the first 24-48 hours, she may never have received the number of antipsychotics that were used by 72 hours into her hospitalization. Furthermore, our case also exposes the common misconception of NMS being a fulminant process; instead, it should be characterized as an insidious process that is often overlooked by even the most senior clinician [3,5].

## Conclusions

NMS is clinically challenging in the setting of cocaine intoxication, as the two can present similarly but require different types of treatment. Furthermore, the practice of the administration of antipsychotic medications for acute cocaine intoxication is called into question here, due to the abrupt dopamine withdrawal that may result in NMS. This theoretical risk may be further increased in cases of long-standing cocaine use, where changes occur over time in the dopamine signaling pathways, causing them to be dysregulated. Further research into this complex clinical situation is needed, as well as a more standardized treatment algorithm for providers managing cocaine-associated agitation in the emergency setting.

## Appendices

A summary of the common clinical findings for NMS and cocaine intoxication is presented in Table 1.

**Table 1:** Clinical considerations for the differentiation of NMS and cocaine intoxication NMS: neuroleptic malignant syndrome

	Neuroleptic malignant syndrome	Acute cocaine intoxication
Onset	1-3 days	Abrupt
Temperature	Increased	Increased
Pupillary size	Normal	Dilated
Muscle tone	Increased	Increased
Peripheral pulses	Normal	Decreased
Pupillary size	Normal	Dilated
Pseudo-hallucinations	Absent	Present
Mental status	Abnormal	Abnormal
Seizures	Absent	Present

Table 2 shows the progression of the patient’s NMS from onset to resolution.

**Table 2:** Trend of objective findings with associated NMS intervention or event ED: emergency department, GMW: general medical ward, ICU: intensive care unit, CPK: creatinine phosphokinase, IU/L: international unit per liter, WBC: white blood cell count, k/uL: thousands per cubic milliliter, °F: temperature in degrees Fahrenheit, RA: room air, FiO2: fraction of inspired oxygen, L: liters, MV: mechanical ventilation, A&O x 4: alert and oriented to person, place, time, and event, NMS: neuroleptic malignant syndrome, CT: computed tomography, MRI: magnetic resonance imaging, LP: lumbar puncture, EEG: electroencephalogram, BFRCS: Bush-Francis Catatonia Rating Scale, SBP: systolic blood pressure, DBP: diastolic blood pressure

	Day #1 (ED)	Day #2 (GMW)	Day #3 (GMW)	Day #4 (GMW)	Day #5 (GMW)	Day #6 (ICU)	Day #7 (ICU)	Day #8 (ICU)	Day #9 (GMW)	Day #10 (GMW)
CPK (IU/L)	-	-	-	3,779	3,159	2,621	1,735	1,393	1,288	776
WBC (k/uL)	7.8	9.5	8.3	22.63	13.6	10.5	12.6	10.3	10.8	9.3
Temperature (°F)	98.8	99.1	97.8	107.2	103.3	101.9	100.2	97.5	98.8	98.3
Heart rate (beats/minutes)	92	97	89	125	117	122	98	104	94	88
Blood pressure (SBP/DBP mmHg)	139/89	133/88	137/83	177/104	155/100	147/99	142/87	138/95	135/87	140/84
Respiratory status	RA	RA	RA	RA	7 (L) FiO2	MV	MV	MV, RA	RA	RA
Mental status	Agitated	Agitated	Agitated	Altered	Increased confusion	-	Follows commands	A&O x 4	Mutism	Volitional mutism
Muscle tone	Normal	Normal	Normal	Normal	Normal	Normal	Normal	Normal	Normal	Normal
Intervention/event	Presents with agitation, received haloperidol 5 mg IV	Admitted to GMW; received quetiapine 200 mg over 16 hours; additional olanzapine, used as needed	Quetiapine 450 mg over 24 hours; additional haloperidol, ziprasidone, used as needed	All antipsychotics stopped; started bromocriptine and lorazepam for NMS; CT and MRI of the head/neck were negative	Neurology consulted; dantrolene added for one dose; intubated later that day	Transfer for ICU level of care; EEG and LP completed	Begins following command; NMS treatment stopped	Extubated	Transfer to GMW	Evaluated for catatonia; BFCRS was 2 and no change with lorazepam challenge; uneventful remainder of hospital course
